# Adapting an Advance Care Planning Website for Persons With Dementia in Nursing Homes During the COVID-19 Pandemic

**DOI:** 10.1093/geroni/igab046.390

**Published:** 2021-12-17

**Authors:** Brianna Morgan, Liza Behrens, Sonia Talwar, Emily Summerhayes, Mary Ersek, Nancy Hodgson

**Affiliations:** 1 University of Pennsylvania, Philadelphia, Pennsylvania, United States; 2 University of Pennsylvania School of Nursing, Philadelphia, Pennsylvania, United States; 3 University of Pennsylvania, School of Nursing, Philadelphia, Pennsylvania, United States; 4 University of Pennsylvania school of nursing, Philadelphia, Pennsylvania, United States; 5 University of Pennsylvania, School of Nursing, philadelphia, Pennsylvania, United States

## Abstract

We partnered with a national for-profit nursing home (NH) organization to test the acceptability and use of an advance care planning (ACP) website for people living with dementia using a randomized controlled trial (RCT) design. Concurrently, the COVID-19 pandemic disproportionately impacted NHs and halted in-person research. We will present challenges, opportunities, and adaptations in site engagement, recruitment, and data collection. Initially, NHs were overwhelmed by pandemic efforts and research staff were unable to enter sites. We capitalized on time and available resources by beta-testing the website in a comparable population and designing surveys to elicit COVID-19’s impact on ACP. Once able, NH staff took on recruitment and data collection efforts intended for research staff. We supported NHs by pivoting to remote data collection, providing technology on site, and offering flexible communication. Flexibility is key in supporting site engagement, recruitment, and data collection and has implications for designing pragmatic RCTs.

